# Associations between childhood overweight, obesity, abdominal obesity and obesogenic behaviors and practices in Australian homes

**DOI:** 10.1186/s12889-017-4595-y

**Published:** 2017-07-21

**Authors:** Seema Mihrshahi, Bradley A. Drayton, Adrian E. Bauman, Louise L. Hardy

**Affiliations:** 1 Prevention Research Collaboration, School of Public Health, The University of Sydney, Charles Perkins Centre D17, Level 6, Sydney, NSW 2006 Australia; 2 NHMRC Centre for Research Excellence in The Early Prevention of Obesity in Childhood, The University of Sydney, Charles Perkins Centre D17, Level 6, Sydney, NSW 2006 Australia; 3 NSW Ministry of Health, NSW Biostatistics Training Program, Sydney, Australia

**Keywords:** Home environment, Parent practices, Weight-related behaviors, Obesity risk, Breakfast, Screen-time

## Abstract

**Background:**

Despite emerging research about the role of the family and home environment on early childhood obesity, little is known on how weight-related behaviors, parent practices and the home environment influence overweight/obesity in older children and adolescents.

**Methods:**

This analysis used data from a cross-sectional, representative population survey of Australian children age 5–16 years conducted in 2015. Data included measured anthropometry to calculate body mass index (BMI; kg/m^2^) and waist-to-height ratio (WHtR; waist circumference/height). Information on home-based weight-related behaviors (individual eating and screen time behaviors, parent influences including rules and home environment factors) were measured using established short questions, with parental proxy reporting for children in up to grade 4, and self-report for students in grades 6, 8 and 10. Logistic regression models were used to examine associations between weight status and home-based weight-related behaviors.

**Results:**

Both children and adolescents who did not consume breakfast daily were more likely to be overweight/obese OR (95% CI) = 1.39 (1.07–1.81) *p* = 0.015, OR (95% CI) =1.42 (1.16–1.74) *p* = 0.001, respectively, adjusted for age, gender, socio-economic status, rural/urban residence and physical activity. There was also a significant positive association with higher waist-to-height ratio in both children and adolescents. Among children, having a TV in the bedroom was also associated with overweight and obesity OR (95% CI) = 1.54 (1.13–2.09) *p* = 0.006 and higher waist-to-height ratio. For adolescents, parenting practices such as having no rules on screen-time, OR (95% CI) = 1.29 (1.07–1.55) *p* = 0.008, and rewarding good behavior with sweets, OR (95% CI) = 2.18 (1.05–4.52) *p* = 0.036, were significant factors associated with overweight and obesity. The prevalence of these obesogenic behaviors were higher in certain sub-groups of children and adolescents, specifically those from social disadvantage and non-English-speaking backgrounds.

**Conclusions:**

Interventions to reduce the prevalence of obesity and overweight should include promoting daily breakfast, reducing screen-time, and encouraging health-promoting parenting practices. Interventions should particularly focus on those at some social disadvantage and from non-English-speaking backgrounds.

## Background

Concern about the high global prevalence of childhood obesity has resulted in considerable research efforts to understand factors associated with unhealthy weight gain [1]. Many behaviors which have been associated with a higher prevalence of obesity, such as poor diet quality, low physical activity and excessive screen-time, track from childhood and adolescence into adulthood [2]. A theoretical framework for understanding the influences of personal and environmental factors that influence behavior is the socio-ecological model (SEM) and this has been used extensively in child obesity research [3].

The SEM comprises five levels of influence, individual, interpersonal, organizational, community, and society and effective prevention and reduction programs should address each of these levels [4]. At an individual and interpersonal level, parents and the home environment are central to the development of a child’s attitudes, beliefs, knowledge and behavior [5]. Understanding eating and activity practices within the home and family environment, which lay the foundation for healthy lifestyles later in life, are fundamental to inform obesity prevention interventions [6].

A child’s home environment is a central influence on their food and eating habits as well as their physical activity and sedentary behavior patterns, particularly screen-time [7] and certain behaviors and environments in the home appear to be more obesogenic than others [8]. For example, eating behaviors known to increase weight status in children and adolescents include skipping breakfast [9], eating dinner in front of the television [10] and eating fast food regularly [11]. Parenting practices such as rewarding children with sweets for good behavior [12] and allowing children unrestricted access to sweet snacks and beverages [13] are known to be associated with increases in obesity. Other parenting practices, such as modeling of good health behaviors, and rule setting with regard to snacking and soft drinks may potentially be protective against overweight and obesity in children [5, 14].

Screen-time (i.e., television, computers, e-devices such as smart phones, tablets) is the most popular sedentary behavior of children and adolescents and high amounts of screen time have been associated with overweight, obesity and poor dietary habits [15]. Parent’s screen-time is known to influence children’s screen-time [16] and there is evidence that homes where there are rules on children’s use of screen devices for recreational purposes provide some protection against overweight and obesity [17].

To date, most interventions to prevent obesity in the home environment have involved children age 0–5 years and are focused on early feeding and parenting [18–20] . While successful child obesity prevention intervention efforts must involve parents during the early stages of child development, the current childhood obesity prevalence suggests the need to provide parents of school age children and adolescents with support to implement healthy practices within the home environment. Identifying parent practices and factors in the home environment that strongly influence childhood overweight/obesity, and the socio-demographic characteristics of children and adolescents at greater risk of engaging in unhealthy weight-related behaviors, can lead to better targeted interventions.

The purpose of this study was to use population health survey data from children age 5–16 years to explore the associations between less healthy individual behaviors, parent influences, the home environment and child and adolescent overweight and obesity. We also examined the associations by socio-demographic characteristics to identify which sub-groups of children are at greater risk of less healthy practices and would benefit most from interventions.

## Methods

This is a secondary data analysis of the 2015 New South Wales (NSW) Schools Physical Activity and Nutrition Survey (SPANS), a representative, cross-sectional school-based surveillance survey of weight and weight-related behaviors of children age 5 to 16 years living in NSW, Australia. The survey was based on a two-stage probability sample (school and student) with the probability of school selection being proportional to size of the school enrollment. Schools were sampled from each education sector (Government, Independent, Catholic) proportional to enrollment in that sector, and then all students from two randomly selected classes in each alternate school grade (Kindergarten, grades 2, 4, 6, 8, 10) were invited to participate. Sample size calculations were based on two primary outcomes: (i) achieving reliable estimates of point prevalence; and (ii) the detection of differences between demographically-defined groups (sub-groups). Sample size was calculated using p_1_ = 0.30 and p_2_ = 0.20 to detect a 10% difference in the prevalence between groups, with 80% power, alpha = 0.05 and design effect was 2.0 indicating 1252 children in each grade group.

The data were collected in schools by trained field teams from February 2015 to April 2015. Informed consent from each child’s parent/carer was a requirement for participation. Ethics approvals were granted by the University of Sydney Human Research Ethics Committee, the NSW Department of Education and Training (DET) and the Catholic Education Offices for the Dioceses of Bathurst, Broken Bay, Canberra, Lismore, Maitland-Newcastle, Parramatta, and Wollongong.

Parents of children in Grades K (Kindergarten) 2 and 4 (i.e. child group) completed the questionnaire at home for their child. Adolescents in grades 6, 8 and 10 (i.e. adolescent group) completed the same questionnaire at school during the field team visit.

### Outcomes

Height (m), weight (kg) and waist circumference (cm) were measured by the trained field teams at school. The Body Mass Index (BMI; kg/m^2^) was calculated and children categorized using the International Obesity Taskforce cut offs as not overweight/obese or overweight/obese [21]. Waist-to-height Ratio (WHtR; cm/cm) was calculated as a measure of abdominal obesity where values ≥0.5 indicate an increased risk of cardio-metabolic outcomes and values <0.5 indicate a low risk for cardio-metabolic outcomes [22].

### Individual behaviors

We assessed four individual behaviors that are associated with child obesity, including the frequency of (i) eating breakfast; (ii) eating dinner in front of the TV; (iii) eating meals or snacks from fast food/takeaway outlets and (iv) using electronic media (e.g. mobile phone or tablet or computer) during sleep time. For the analyses, each factor was dichotomized; breakfast as daily or not daily, eating dinner in front of the TV as <5 or ≥5 times/week, fast food consumption as <1 or ≥1 time/week and using e-media never/sometimes or usually.

### Parenting influences

We assessed two parenting practices associated with child obesity using validated questions from the Child Feeding Questionnaire [23]. This included asking about (i) ad libitum snacking (i.e., *At home, does your child/do you snack (on chips, biscuits, muesli bars* etc.*) and or drink soft drink whenever they like?* (response categories; Yes or No, they/I have to ask first), and (ii) using food as a reward (i.e., *How often do you/your parents offer your child/you sweets (lollies, ice cream, cake, biscuits) to your child as a reward for good behavior?* (response categories; never/rarely, sometimes, usually). For the analyses, responses for this question were dichotomized as ‘never/sometimes’ and ‘usually’. The following question was used to determine how often limits were set on screen time, *How often do you/your parents set limits/rules on your child’s/your screen-time (*e.g.*, TV, DVDs, electronic games, tablets, mobile/smart phone)?* (response categories; never/rarely, sometimes, usually). For the analyses, responses were dichotomized as ‘never/sometimes’ and ‘usually’.

### Home environment factors

We assessed two factors in child’s homes that are consistently associated with overweight/obesity. The first was (i) *Does your child/you have a TV in the bedroom?* (response categories Yes or No). The second, related to the availability of soft drinks (sugar-sweetened beverages) in the home, was assessed by asking *How often are soft drinks available in your home? (*response categories never, sometimes, usually). For the analyses, responses were dichotomized as ‘never/sometimes’ and ‘usually’.

### Covariates

Demographic information included child’s sex, date of birth, language spoken most at home and postcode of residence. Postcode of residence was a proxy measure of socioeconomic status (SES) using the Australian Bureau of Statistics’ Socioeconomic Index for Areas (SEIFA) Index of Relative Socioeconomic Disadvantage [24]. SEIFA summarizes census-obtained socioeconomic indicators for geographic areas including income, educational attainment, unemployment, and the proportion of people in unskilled occupations and were used to rank students into low, middle, and high tertiles of SES. Postcode was also used to categorize whether the child resided in a rural or urban location [25]. Language spoken most often at home was used to categorize children into English-speaking or non-English-speaking background [26]. Physical activity participation (a potential confounder) was determined using a validated question that is recommended for health surveillance surveys [27] which asks: *How many days did the child/you engage in moderate-to-vigorous physical activity for at least 60-min each day in the past 7-days?* [28]. Physical activity participation was dichotomized according to the recommendation, <7 or 7-days (i.e., met recommendations or did not meet recommendations).

### Statistical analysis

Descriptive statistics and logistic regression analyses were conducted using SPSS Complex Sample Analysis (Version 22 for Windows; IBM, Chicago, IL, USA) to account for the complex sampling design. Post stratification weights were used to account for variations in response rates, along with cluster and stratification variables to account for the complex sampling design. Analyses were stratified by method of survey completion i.e. proxy report by parents for children in Grades K, 2 and 4 (child group) and self-report for adolescents in Grades 6, 8 and 10 (adolescent group). For the logistic regression analyses variables that showed significance (*p* < 0.05) on univariate analysis were entered into the model and a forward stepwise procedure was used to check the effect on the β coefficient. The final model was adjusted for age, sex, SES tertile, residential location (urban/rural), cultural background (English-speaking/other) and meeting daily physical activity recommendations (60 mins daily) or not.

## Results

The sample comprised 3884 children (response rate = 68%; mean age 7.3 years) and 3671 adolescents (response rate = 51%; mean age 13.4 years) from 84 schools. The sample characteristics are shown in Table 1 and indicate there were no socio-demographic differences between child and adolescent groups, however the odds of being overweight/obese were 32% higher among adolescents, compared with children (OR 1.32 95% CI: 1.11, 1.58).

**Table 1:** Characteristics of the sample, stratified by age groups (*n* = 7555)^a^

Characteristic	n	Children (Grades K, 2, 4)	n	Adolescents (Grades 6, 8, 10)	*P*-value
Age (years, mean, SE)	3884	7.27 (0.03)	3671	13.44 (0.04)	
Boys (%, SE)	1826	49.0 (1.3)	1837	50.4 (3.0)	0.657
Weight status indicators (%, SE)					
Overweight/obese^b^	828	21.9 (1.6)	995	27.0 (1.3)	0.003
WHtR ≥ 0.5	542	14.5 (1.4)	471	13.2 (1.2)	0.424
Socio-economic tertile (%, SE)					
Low	808	21.8 (5.8)	1039	31.3 (4.4)	0.211
Middle	1303	33.6 (6.7)	1298	33.2 (4.1)	
High	1773	44.6 (7.3)	1334	35.4 (4.7)	
Cultural background (%, SE)					
English-speaking	3320	87.2 (2.5)	3181	87.5 (2.0)	0.910
Non-English speaking	489	12.8 (2.5)	450	12.5 (2.0)	
Residential location (%, SE)					
Urban	2991	78.6 (6.6)	2781	73.9 (5.0)	0.475
Rural	893	21.4 (6.6)	890	26.1 (5.0)	

*WHtR* waist-to-height ratio

^a^weighted proportions given

^b^using International Obesity Task Force cut-offs [21]

**Fig. 1:**
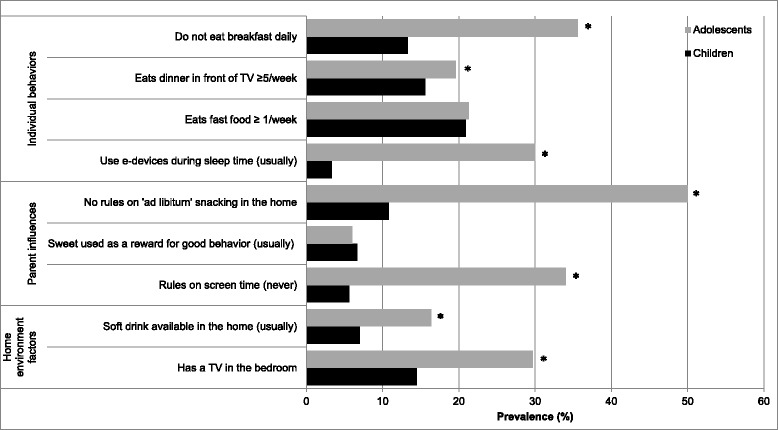
Prevalence of individual behaviors, parent influences, and home environment factors, by age group (%)

Figure 1 shows the prevalence of weight-related individual behaviors, parent influences and home environment factors related to diet and screen-time, stratified by child and adolescent group. The figure shows that as children shift into adolescence there are clear and significant differences, with behaviors such as not eating breakfast daily (*p* < 0.001), having dinner in front of the TV ≥ 5 times week (*p* < 0.001) and using e-media during sleep time (*p* < 0.001) being more prevalent in adolescents. Compared with children, adolescents were more likely to have less parental restriction on snacking in the home (*p* < 0.001) and screen-time (*p* < 0.001), and to usually have soft drinks in the home (*p* < 0.001) and have a TV in the bedroom (*p* < 0.001) .

Table 2 shows the associations between individual behaviors, parent influences, and home environment and unhealthy weight status in children. The univariate analysis showed that not eating breakfast daily, eating fast food more than once a week, usually having soft drink (sugar sweetened beverages) available in the home, and having a TV in the bedroom were risk factors for overweight/obesity. After adjustment for covariates the odds of being overweight/obese were estimated to be 1.39 times higher for children who did not eat breakfast daily (*p* = 0.015), compared with children who ate breakfast daily and estimated to be 1.54 times higher if they had a TV in the bedroom (*p* = 0.006), compared with children who did not have TV in the bedroom. The same behavioral and home environment factors were associated with abdominal obesity in children (i.e., WHtR ≥0.5) but also included using e-media during sleep time and having no rules on snacking at home or screen-time. Once adjusted for covariates, the odds of having abdominal obesity were estimated to be 1.67 times higher for children who did not eat breakfast daily, compared with children who ate breakfast daily (*p* = 0.002), 1.67 times higher if they never had rules on screen-time (*p* = 0.007), and 1.80 times higher if they had a TV in the bedroom, compared with children who did not have TV in the bedroom (*p* = 0.001).

**Table 2:** Association between individual behaviors, parent influences, and home environment factors and overweight/obesity and WHtR ≥ 0.5 in children (*n* = 3884)

	Overweight/obese				WHtR ≥ 0.5			
Child Behaviors	OR (95% CI)	*P* value	AOR^a^ (95% CI)	*P* value	OR (95% CI)	*P* value	AOR^a^ (95% CI)	*P* value
Does eat breakfast daily	1.0				1.0		1.0	
Do not eat breakfast daily	1.58 (1.18, 2.11)	0.001	1.39 (1.07, 1.81)	0.015	1.94 (1.36, 2.76)	0.001	1.67 (1.20, 1.33)	0.002
Eats dinner in front of TV <5/week	1.0				1.0		1.0	
Eats dinner in front of TV ≥5/week	1.24 (0.91, 1.68)	0.160	1.19 (0.87, 1.62)	0.270	1.33 (0.93, 1.91)	0.114	1.18 (0.78, 1.77)	0.422
Eats fast food <1/week	1.0				1.0		1.0	
Eats fast food ≥1/week	1.37 (1.10, 1.70)	0.005	1.23 (0.98, 1.56)	0.074	1.44 (1.15, 1.82)	0.001	1.30 (0.99, 1.68)	0.055
Use e-devices during sleep time (never/sometimes)	1.0				1.0		1.0	
Use e-devices during sleep time (usually)	1.26 (0.78, 2.05)	0.347	1.08 (0.68, 1.73)	0.731	1.62 (1.00, 2.62)	0.045	1.50 (0.88, 2.38)	0.136
Parent influences								
Rules on snacking in the home	1.0				1.0		1.0	
No rules on snacking in the home	1.15 (0.84, 1.57)	0.389	1.16 (0.88, 1.53)	0.276	1.46 (1.05, 2.03)	0.022	1.20 (0.88, 1.63)	0.246
Sweet used as a reward for good behavior (never/sometimes)	1.0				1.0		1.0	
Sweet used as a reward for good behavior (usually)	0.96 (0.71, 1.31)	0.810	1.02 (0.76, 1.38)	0.880	1.01 (0.64, 1.60)	0.965	0.90 (0.56, 1.44)	0.648
Rules on screen-time (usually)	1.0				1.0		1.0	
Rules on screen-time (never/sometimes)	1.20 (0.85, 1.69)	0.310	1.22 (0.85, 1.75)	0.268	1.70 (1.19, 2.43)	0.003	1.67 (1.14, 2.45)	0.007
Home environment factors								
Soft drink available in the home (never/sometimes)	1.0				1.0		1.0	
Soft drink available in the home (usually)	1.51 (1.13, 2.01)	0.005	1.24 (0.94, 1.64)	0.134	1.50 (1.07, 2.11)	0.017	1.25 (0.85, 1.84)	0.248
No TV in the bedroom	1.0				1.0		1.0	
Has a TV in the bedroom	1.74 (1.28, 2.36)	0.001	1.54 (1.13, 2.09)	0.006	1.96 (1.47, 2.61)	0.001	1.80 (1.31, 2.46)	0.001

*WHtR* waist-to-height ratio

^a^adjusted for age, sex, socio-economic status, rural/urban residence, cultural background and meeting daily physical activity recommendation

Table 3 shows the associations between individual behaviors, parent influences, and home environment and unhealthy weight status in adolescents. Factors which were significantly associated with being overweight/obese at a univariate level remained significant following adjustment of covariates. The adjusted odds of being overweight/obese were estimated to be 1.42 times higher for adolescents who do not eating breakfast daily (*p* = 0.001), 1.30 times higher if screen devices were usually used during sleep time (*p* = 0.022), 2.18 times higher when parents usually reward for good behavior with sweets (*p* = 0.036), and 1.29 higher when there were no rules on screen-time (*p* = 0.008). In contrast to children, only two factors were associated with abdominal obesity in adolescents at the univariate level, (not eating breakfast daily and having a TV in the bedroom) and once adjusted for covariates, not eating breakfast daily was the only significant risk factor (OR 1.37, *p* = 0.007).

**Table 3:** Association between individual behaviors, parent influences, and home environment factors and overweight/obesity and WHtR ≥ 0.5 in adolescents (*n* = 3671)

	Overweight/obese				WHtR ≥ 0.5			
Adolescent behaviors	OR (95% CI)	*P* value	AOR^a^ (95% CI)	*P* value	OR (95% CI)	*P* value	AOR^a^ (95% CI)	*P* value
Does eat breakfast daily	1.0				1.0		1.0	
Do not eat breakfast daily	1.51 (1.25, 1.83)	0.001	1.42 (1.16, 1.74)	0.001	1.42 (1.15, 1.77)	0.001	1.37 (1.09, 1.73)	0.007
Eats dinner in front of TV <5/week	1.0				1.0		1.0	
Eats dinner in front of TV ≥5/week	1.08 (0.88, 1.34)	0.464	1.02 (0.83, 1.27)	0.839	1.18 (0.89, 1.56)	0.234	1.06 (0.79, 1.43)	0.69
Eats fast food less than once/week	1.0				1.0		1.0	
Eats fast food ≥1/week	0.96 (0.78, 1.19)	0.742	0.88 (0.71, 1.09)	0.228	1.08 (0.83, 1.40)	0.558	0.93 (0.72, 1.34)	0.962
Use e-devices during sleep time (never/sometimes)	1.0				1.0		1.0	
Use e-devices during sleep time (usually)	1.29 (1.04, 1.59)	0.021	1.30 (1.04, 1.63)	0.022	1.14 (0.86, 1.51)	0.358	1.20 (0.86, 1.67)	0.272
Parent practices								
Rules on snacking in the home	1.0				1.0		1.0	
No rules on snacking in the home	0.90 (0.73, 1.11)	0.322	0.85 (0.69, 1.04)	0.103	1.90 (0.67, 1.20)	0.465	0.83 (0.61, 1.13)	0.239
Sweet used as a reward for good behavior (never/sometimes)	1.0				1.0		1.0	
Sweet used as a reward for good behavior (usually)	1.85 (1.04, 3.30)	0.035	2.18 (1.05, 4.52)	0.036	0.73 (0.33, 1.60)	0.419	0.63 (0.23, 1.68)	0.351
Rules on screen-time (usually)	1.0				1.0		1.0	
Rules on screen-time (never/sometimes)	1.24 (1.03, 1.51)	0.027	1.29 (1.07, 1.55)	0.008	1.17 (0.95, 1.43)	0.126	1.21 (0.97, 1.51)	0.086
Home environment								
Soft drink available in the home (never/sometimes)	1.0				1.0		1.0	
Soft drink available in the home (usually)	1.23 (0.98, 1.55)	0.068	1.16 (0.92, 1.46)	0.222	1.07 (0.80, 1.45)	0.636	0.99 (0.71, 1.37)	0.941
No TV in the bedroom	1.0				1.0		1.0	
Has a TV in the bedroom	1.23 (0.99, 1.54)	0.063	1.23 (0.98, 1.55)	0.071	1.38 (1.02, 1.86)	0.036	1.20 (0.90, 1.60)	0.216

*WHtR* waist-to-height ratio

^a^adjusted for age, sex, socio-economic status, rural/urban residence, cultural background and meeting daily physical activity recommendation

The adjusted logistic regression models for predicting obesity/overweight measured by BMI and abdominal obesity measured by WHtR in children and adolescents are shown in Table 4. Among children, not eating breakfast daily and having a TV in the bedroom were the most significant predictors for both overweight/obesity and abdominal obesity. An additional predictor, not having limits on screen-time was a significant predictor of abdominal obesity in children. For adolescents, in addition to not eating breakfast daily, parenting practices such as rewarding good behavior with sweets and no rules on screen-time were significant predictors of obesity/overweight measured by BMI. For abdominal obesity in adolescents the only significant predictor was not eating breakfast daily.

**Table 4:** Adjusted logistic regression models for predicting overweight and obesity (BMI) and abdominal obesity (WHtR ≥ 0.5) in children and adolescents

Children			Adolescents		
Obesity/overweight (BMI)	AOR^a^ (95% CI)	*P* value		AOR^a^ (95% CI)	*P* value
Does not eat breakfast daily	1.36 (1.01–1.71)	0.038	Does not eat breakfast daily	1.42 (1.16–1.74)	0.001
TV in the bedroom	1.52 (1.12–2.08)	0.007	Sweet used as a reward for good behavior	2.36 (1.14–4.84)	0.02
			No screen time rules	1.25 (1.04–1.50)	0.01
Abdominal obesity (WHtR ≥ 0.5)					
Does not eat breakfast daily	1.54 (1.06–2.22)	0.021	Does not eat breakfast daily	1.37 (1.09–1.73)	0.007
No screen time rules	1.66 (1.16–2.38)	0.005			
TV in the bedroom	1.75 (1.26–2.43)	0.001			

*WHtR* waist-to-height ratio

^a^adjusted for age, sex, socio-economic status, rural/urban residence, cultural background, meeting daily physical activity recommendation and other variables listed in the model

An analysis of the significant factors was undertaken by socio-demographic sub-groups (data not shown). Children who do not eat breakfast daily were more likely to: be girls, compared with boys (OR 1.27 95% CI: 1.03, 1.57); come from low, compared with high SES neighborhoods (OR 2.82 95% CI: 1.73, 4.60); come from non-English-speaking, compared with English-speaking cultural backgrounds (OR 3.07 95% CI: 2.22, 4.25); and live in urban, compared with rural areas (OR 1.68 95% CI: 1.14, 2.47). Children with TV’s in the bedroom were more likely to come from low (OR 3.39 95% CI: 2.21, 5.20) and middle (OR 2.50 95% CI: 1.59, 3.94), compared with high SES neighborhoods; and girls were less likely to have screen-time rules imposed (OR 0.71 95% CI: 0.51, 0.99), compared with boys.

Adolescents who do not eat breakfast daily were more likely to be girls (OR 1.35 95% CI: 1.12, 1.63), come from low SES (OR 1.84 95% CI: 1.43, 2.37) and middle (OR 1.33 95% CI: 1.03, 1.73) SES neighborhoods, compared with high SES peers and come from non-English speaking, compared with English-speaking cultural backgrounds (OR 1.99 95% CI: 1.15, 3.43). Adolescents with TV’s in the bedroom were more likely to be boys (OR 2.09 95% CI: 1.64, 2.65), come from low (OR 2.11 95% CI: 1.49, 3.00) and middle (OR 1.87 95% CI: 1.31, 2.67), compared with high SES neighborhoods, come from English-speaking, compared with non-English speaking backgrounds (OR 2.15 95% CI: 1.52, 3.04) and live in urban, compared with rural areas (OR 1.54 95% CI: 1.06, 2.23). Adolescents from rural areas were more likely to never have screen-time rules imposed, compared with adolescents from urban areas (OR 1.31 95% CI: 1.02, 1.69); and adolescents with parents who reward their good behavior with sweets are more likely to be girls (OR 1.54 95% CI: 1.04, 2.28) and to come from non-English speaking backgrounds, compared with English-speaking cultural backgrounds (OR 1.73 95% CI: 1.16, 2.57).

## Discussion

Our findings were based on a large representative sample of children and adolescents and show there are behaviors and practices within the home environment that are strongly associated with overweight/obesity and abdominal obesity in children and adolescents. Identifying these behaviors and practices and understanding how they are influenced in the family context is essential for the design of successful interventions. As with other studies, our findings show that the prevalence of many of these individual behaviors and practices is higher among adolescents [29, 30]. This is likely to reflect the increased autonomy during adolescence [31], however, prospective studies are required to ascertain whether exposure to, and adoption of these behaviors and practices during childhood influences their maintenance in adolescence. The findings illustrate that different family-based intervention strategies are needed for children and for adolescents, and these strategies are best directed at specific sub-groups of children and adolescents, specifically those from social disadvantage and non-English-speaking backgrounds.

A novel aspect of our study was to include abdominal obesity as an outcome. Compared with BMI measures of generalized obesity, abdominal obesity is more strongly correlated with metabolic risk factors [32] with evidence showing that abdominal obesity as indicated by WHtR ≥ 0.5 is a strong predictor of cardio-metabolic risk in children [33]. Although the prevalence of abdominal obesity in our study (13–14%) is much lower than US prevalence rates (~30–36%) [34] there is evidence that abdominal obesity has increased over the last 30-years in Australian children and adolescents [35] and warrants on-going surveillance given the associated cardio-metabolic risks. Interestingly, we found that not having breakfast daily and having a TV in the bedroom were the strongest predictors of both BMI measured overweight/obesity (using kg/m^2^) and abdominal obesity (using WHtR ≥0.5) in children. Among adolescents, not eating breakfast daily was the strongest predictor of overweight and obesity and abdominal obesity.

Our results are similar to other studies which show the importance of breakfast consumption [36] [9]. Recent data from the International Study of Childhood Obesity, Lifestyle and the Environment (ISCOLE) showed that more frequent breakfast consumption was associated with lower BMI z-scores and body fat percentage compared with occasional and rare consumption [9]. The proposed mechanism for the protective effect of eating breakfast on obesity and overweight is that it may reduce snacking and consumption of energy-dense nutrient-poor foods later in the day [37]. Skipping breakfast may also have long term effects. In a longitudinal Australian study of children age 9–15 years over 20 years, participants who reported skipping breakfast in both childhood and adulthood had larger waist circumferences, higher BMI, and poorer cardio-metabolic profiles than did those who reported eating breakfast at both time points [38]. A recent review of longitudinal studies also found similar results [39] and there may be other reasons to implement interventions to improve breakfast intake in children, beyond obesity and overweight, as an increased frequency of breakfast consumption has been consistently associated with improved academic performance [40]. International data also suggests that the prevalence children and adolescents consuming breakfast daily remains low [41], indicating the need for effective interventions to encourage daily healthy breakfasts and promote nutritious choices in these age groups.

In addition to not eating breakfast daily, having a TV in the bedroom was a major factor associated with overweight and obesity among children, and for adolescents not having limits set on screen-time was a significant factor. The reason may be two fold; children who have higher screen-time do less physical activity [42–44], and TV and internet food and beverage advertising has an influence on children’s and adolescent’s food choices [45]. Many studies have shown that excessive TV watching may favor concurrent consumption of energy-dense snacks and beverages [46, 47]. More frequent TV dinners were associated with more frequent consumption of soft drinks and snacks [48]. Screen-time has also been associated with obesity in both cross-sectional [49] and longitudinal studies [15]. Parenting practices can either facilitate or inhibit healthy eating and many studies have associated various parenting practices and styles with obesity and overweight [50] [12]. Rewarding good behavior with sweets suggests a permissive parenting style that may be a risk factor for obesity [51].

While our study had strengths including two outcome measures of unhealthy weight status and the large representative sample size of children and adolescents, there are limitations to consider when interpreting our findings. Because the data are cross-sectional no firm causal relationships can be determined. However, observational studies are an important information source for identifying sub-populations at greatest risk of poor health outcomes and would benefit most from health promoting interventions. Each construct was assessed by one question, and as such there may have been some reporting problems including recall issues and social desirability bias. Another limitation was the difference in the responder between the child and adolescent groups. Parents responded to the questionnaire on behalf of children and self-report measures were collected for adolescents. Due to these differences in responder, children and adolescents cannot strictly be compared.

## Conclusions

Although interventions across multiple settings are required to address the rising prevalence of childhood obesity, the home is potentially the most important setting. The family and home environment are a major influence on child and adolescents eating and other lifestyle behaviors –not only because parents provide the food and environments, but the whole family influences attitudes, preferences and values that affect lifetime habits. Our findings suggest that four specific modifiable weight-related behaviors and practices that occur in the home; skipping daily breakfast, having a TV in the bedroom, not imposing rules on children and adolescent’s screen-time and rewarding adolescent’s good behavior with sweets are important factors to consider in home-based child obesity prevention interventions. Simple strategies such as reinforcing the importance of nutritious breakfasts and encouraging removal of TV’s from children’s bedrooms may be implemented immediately. Our findings also show that these behaviors and practices differ across sub-group populations and parents of children and adolescents from low SES neighborhoods and non-English-speaking backgrounds in particular would benefit from interventions that support changing these behaviors.

## Abbreviations

BMI: Body Mass Index
NSW: New South Wales
SEIFA: Socioeconomic Index for Areas
SES: Socioeconomic Status
SPANS: School Physical Activity and Nutrition Survey
TV: Television
WHtR: Waist-to-height ratio
